# Supplementary material for: Longitudinal profiling of the intestinal microbiome in children with cystic fibrosis treated with elexacaftor-tezacaftor-ivacaftor
Source: mBio. 2024 Jan 26;15(2):e01935-23. doi: 10.1128/mbio.01935-23 (PMC10865789; doi:10.1128/mbio.01935-23)
Supplement: Supplemental Figures — Figures S1-S7. [file mbio.01935-23-s0002.pdf]

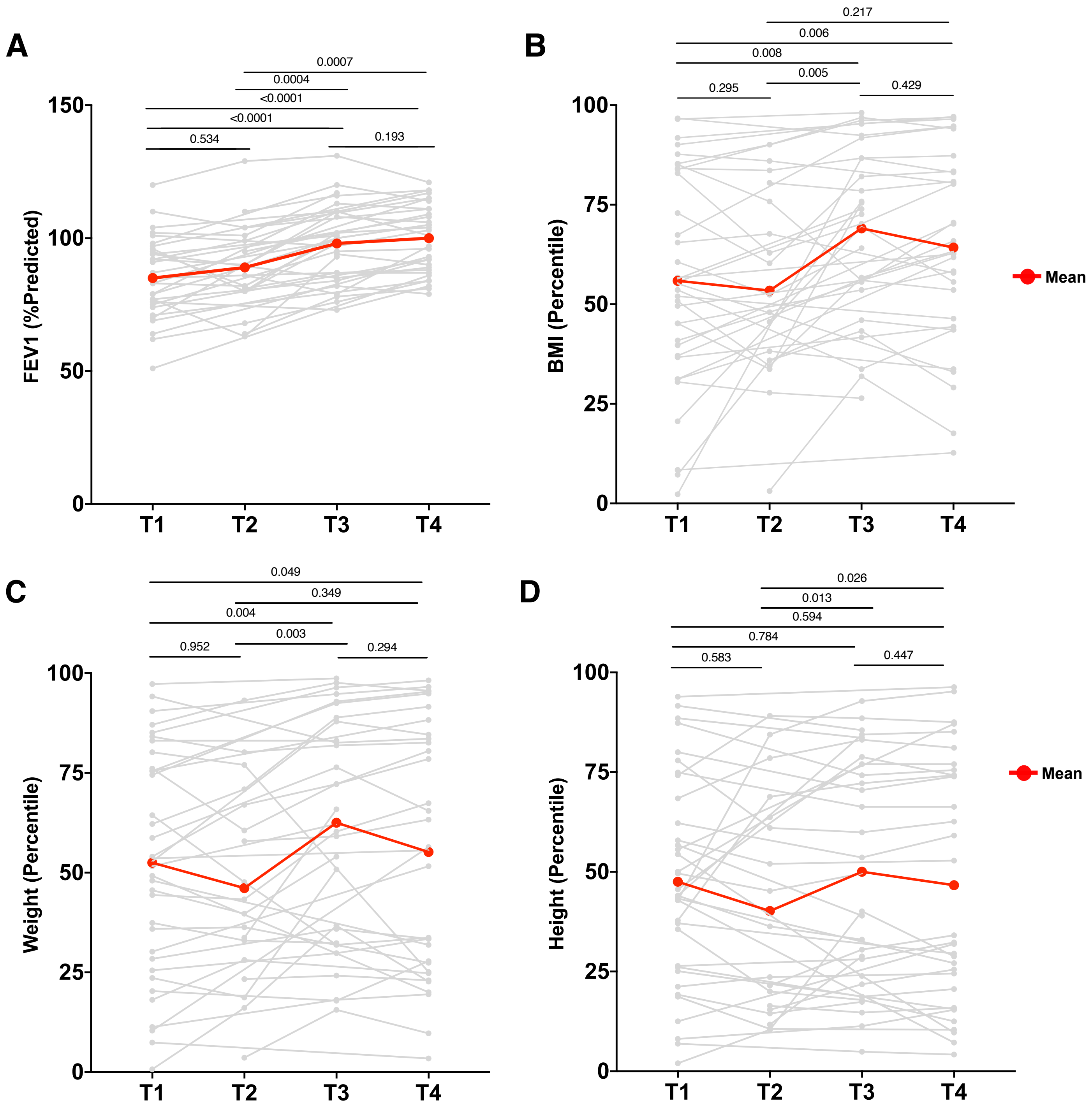

Supplementary Figure S1. A-D) Clinical metadata vs. timepoint; **(A)** ppFEV1, **(B)** BMI percentile, **(C)** weight percentile, and **(D)** height percentile. Each dot represents the clinical data associated with a stool sample. Red line indicates the mean, and gray lines represent individual patients. P values calculated by Wilcoxon signed-rank test.

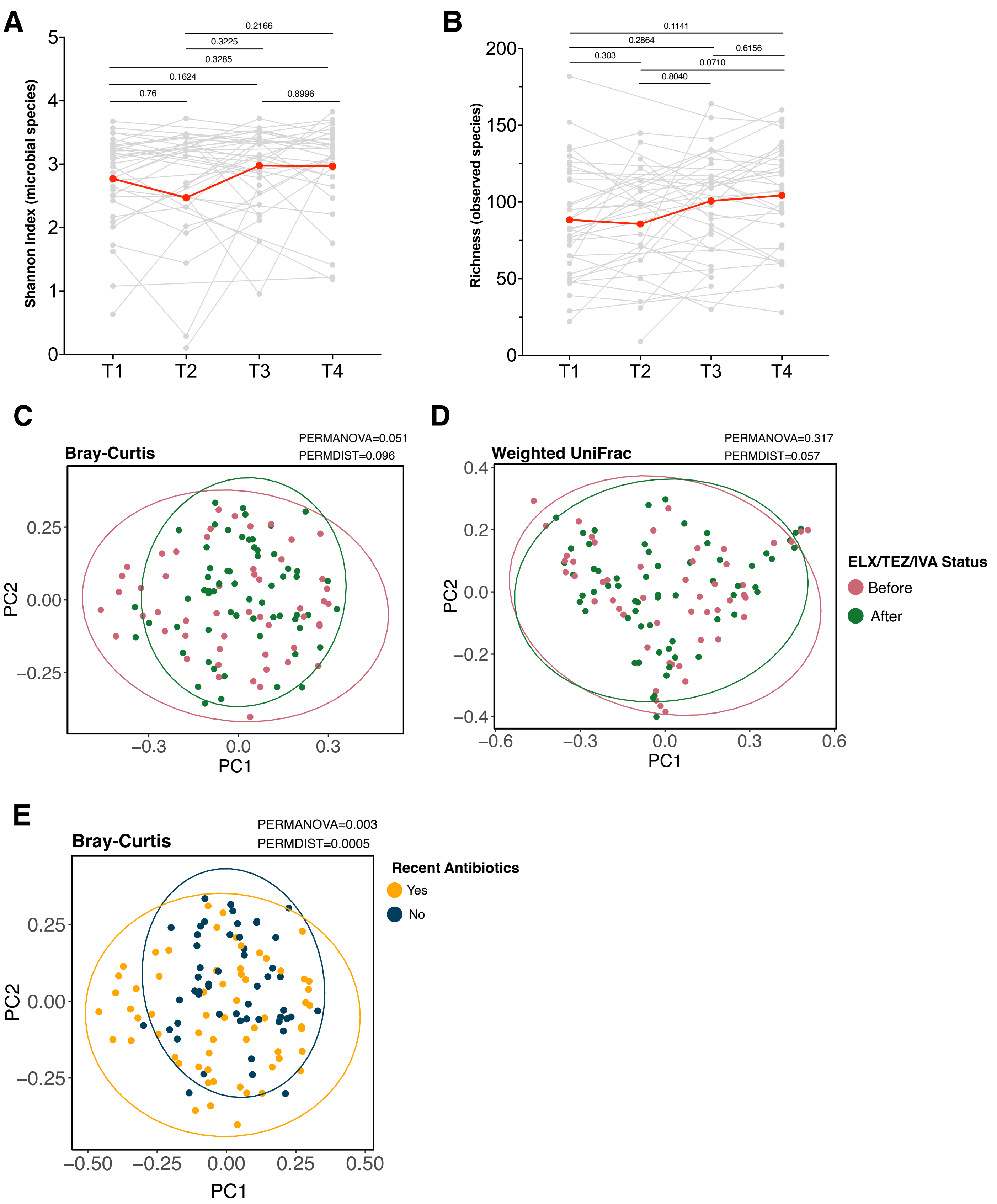

Supplementary Figure S2. A-B) Alpha diversity vs. timepoint. Shannon Index (**A**) calculated with the R package vegan using species abundance table from Metaphlan4. Microbial richness (**B**) represents the number of unique species per sample. Each dot represents a stool sample (samples=114). P values calculated by Wilcoxon signed-rank test. **C)** Principal component analysis of Bray-Curtis distances before and after

ELX/TEZ/IVA. Bray-Curtis distance matrix computed with vegan. PERMANOVA and PERMDISP computed by the vegan package with the functions adonis2 and betadisp, respectively. Ellipse depicts the 95% confidence level. **D)** Principal component analysis of weighted UniFrac distances before and after ELX/TEZ/IVA. Weighted UniFrac computed with MetaPhlAn4 R script. PERMANOVA and PERMDISP computed by the vegan package with the functions adonis2 and betadisp, respectively. Ellipse depicts the 95% confidence level. **E)** Principal component analysis of the Bray-Curtis distances between samples with and without recent antibiotic exposure. Distance matrix generated by the R package vegan using the MetaPhlAn4 species table. PERMANOVA and PERMDISP computed in vegan with the functions adonis2 and betadisp, respectively. Ellipse depicts the 95% confidence level.

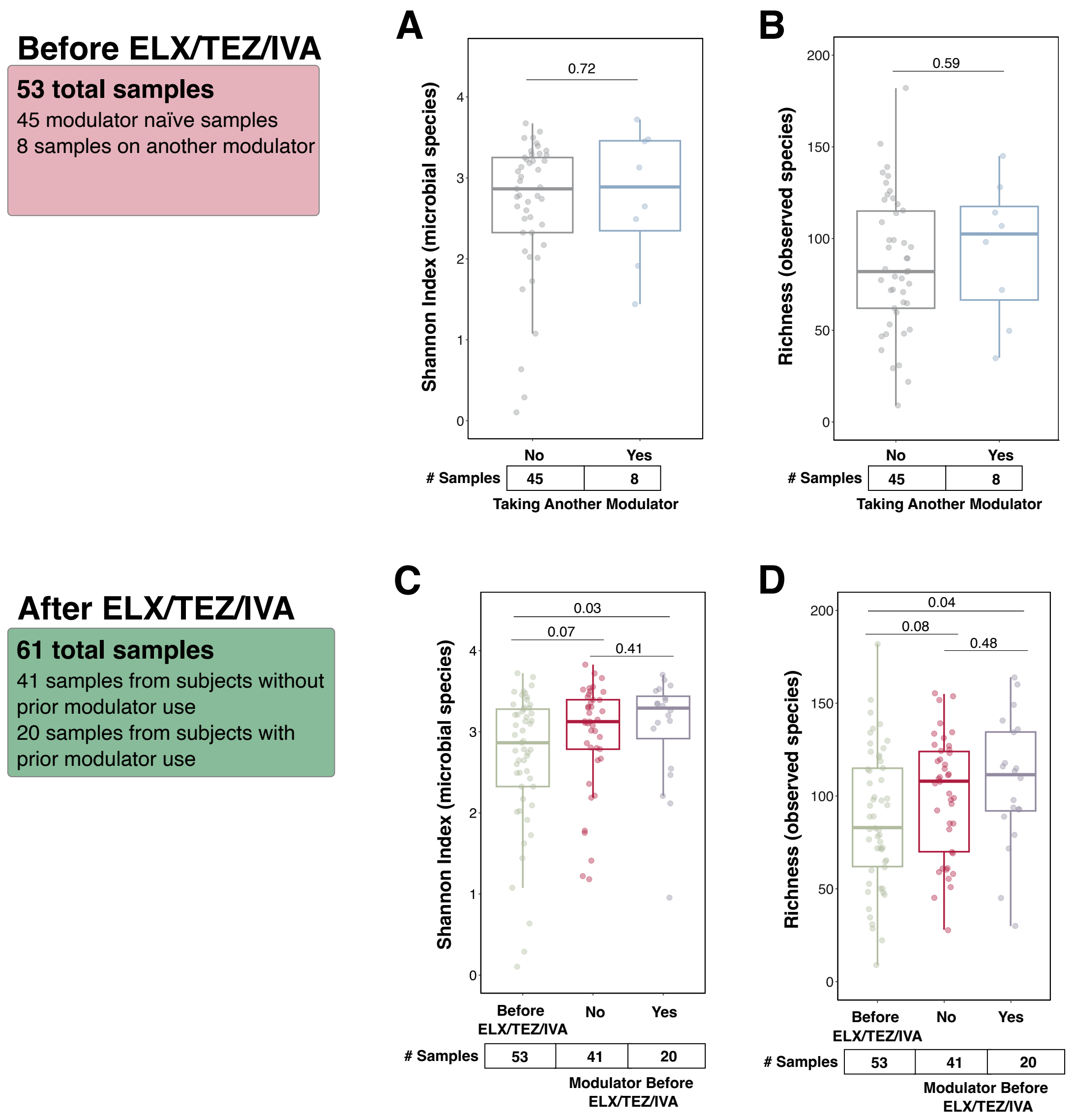

Supplementary Figure S3. A-B) Diversity metrics of pre-ELX/TEZ/IVA samples (samples=53 total). Samples are grouped by whether the subject was receiving another CFTR modulator at the time of sample collection (Yes=8, No=45). **C-D)** Diversity metrics of pre-ELX/TEZ/IVA samples (samples=53) compared to post-ELX/TEZ/IVA samples (samples=61). Post-ELX/TEZ/IVA samples were segregated by whether the subject had previously received any other CFTR modulator (Yes=20 samples, No=41 samples). Shannon Index calculated with the R package vegan using species abundance table from MetaPhlAn4. Microbial richness represents the number of unique species per sample. P values calculated by Wilcoxon rank-sum test.

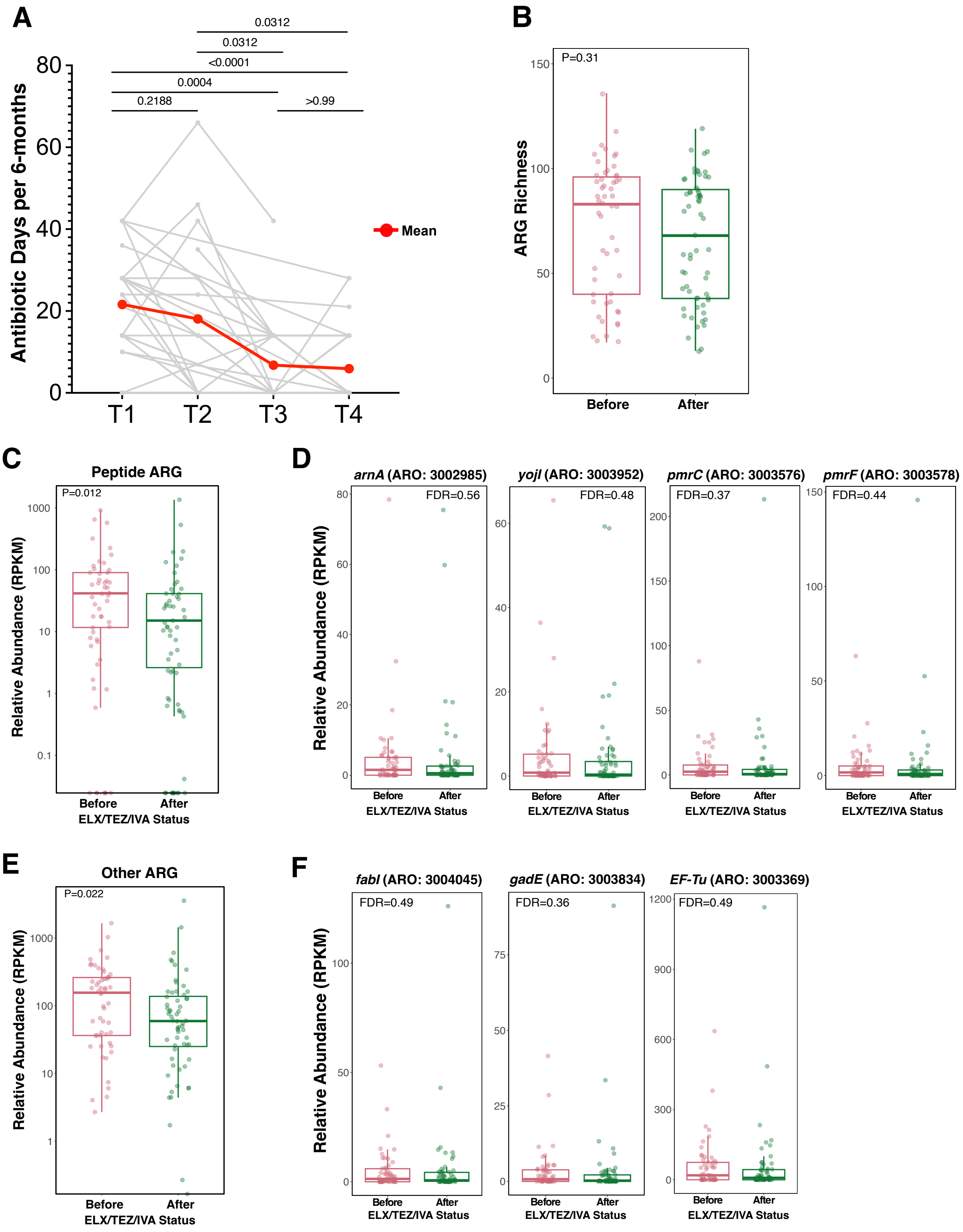

Supplementary Figure S4. A) Cumulative antibiotic days per 6-months prior to stool sample collection. P values calculated by Wilcoxon signed-rank test. **B)** ARG richness (unique genes) before and after ELX/TEZ/IVA. ARGs were profiled using ShortBRED and the Comprehensive Antibiotic Resistance Database (CARD). Each dot represents a stool sample (samples=114). P values calculated by Wilcoxon rank-sum test. **C)** Cumulative relative abundance (RPKM) of ARGs conferring resistance to peptide antibiotics. P values calculated by Wilcoxon rank-sum test. **D)** Relative abundance (RPKM) of the most prevalent ARGs conferring

resistance to peptide antibiotics. MaAsLin2 FDR is depicted. **E)** Cumulative relative abundance (RPKM) of ARGs conferring resistance to other antibiotics. P values calculated by Wilcoxon rank-sum test. **F)** Relative abundance (RPKM) of the most prevalent ARGs conferring resistance to other antibiotics. MaAsLin2 FDR is depicted.

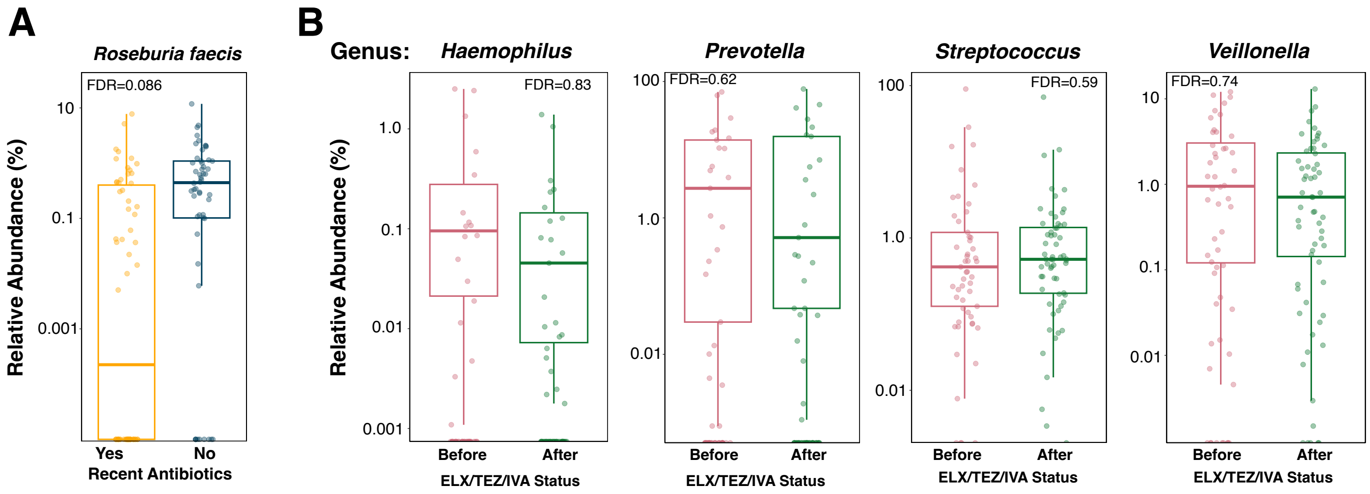

Supplementary Figure S5. A) *Roseburia faecis* relative abundance between samples with and without recent antibiotic exposure. Each dot represents a stool sample (samples=114). MaAsLin2 FDR is depicted. **B)** Relative abundance of respiratory genera in stool samples before and after ELX/TEZ/IVA. Each dot represents a stool sample (samples=114). MaAsLin2 FDR is depicted. Vertical axis is log10 transformed. Zero values are plotted on the horizontal axes.

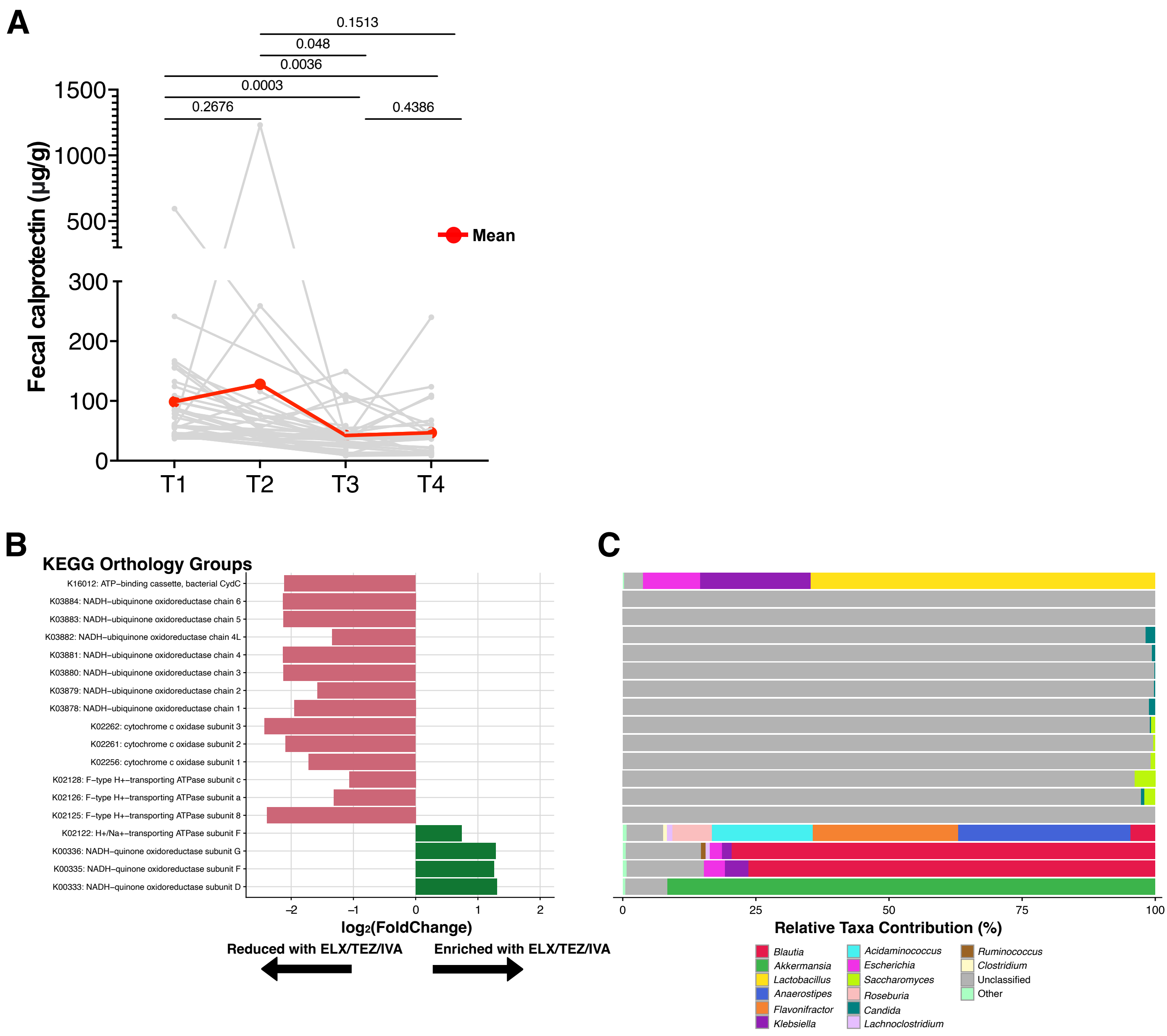

Supplementary Figure S6. A) Fecal calprotectin (µg/g) vs. study timepoint. Red line indicates the mean, and gray lines represent individual patients. P values calculated by Wilcoxon signed-rank test. **B)** Differentially abundant oxidative phosphorylation KEGG orthology (KO) groups from MaAsLin2 (FDR<0.25). Groups correspond to red points in Fig. 5A. log2(FoldChange), which is equivalent to MaAsLin2 coefficient, is depicted. **C)** Taxonomic stratification of differentially abundant oxidative phosphorylation KO groups. KO groups correspond to groups in panel A.

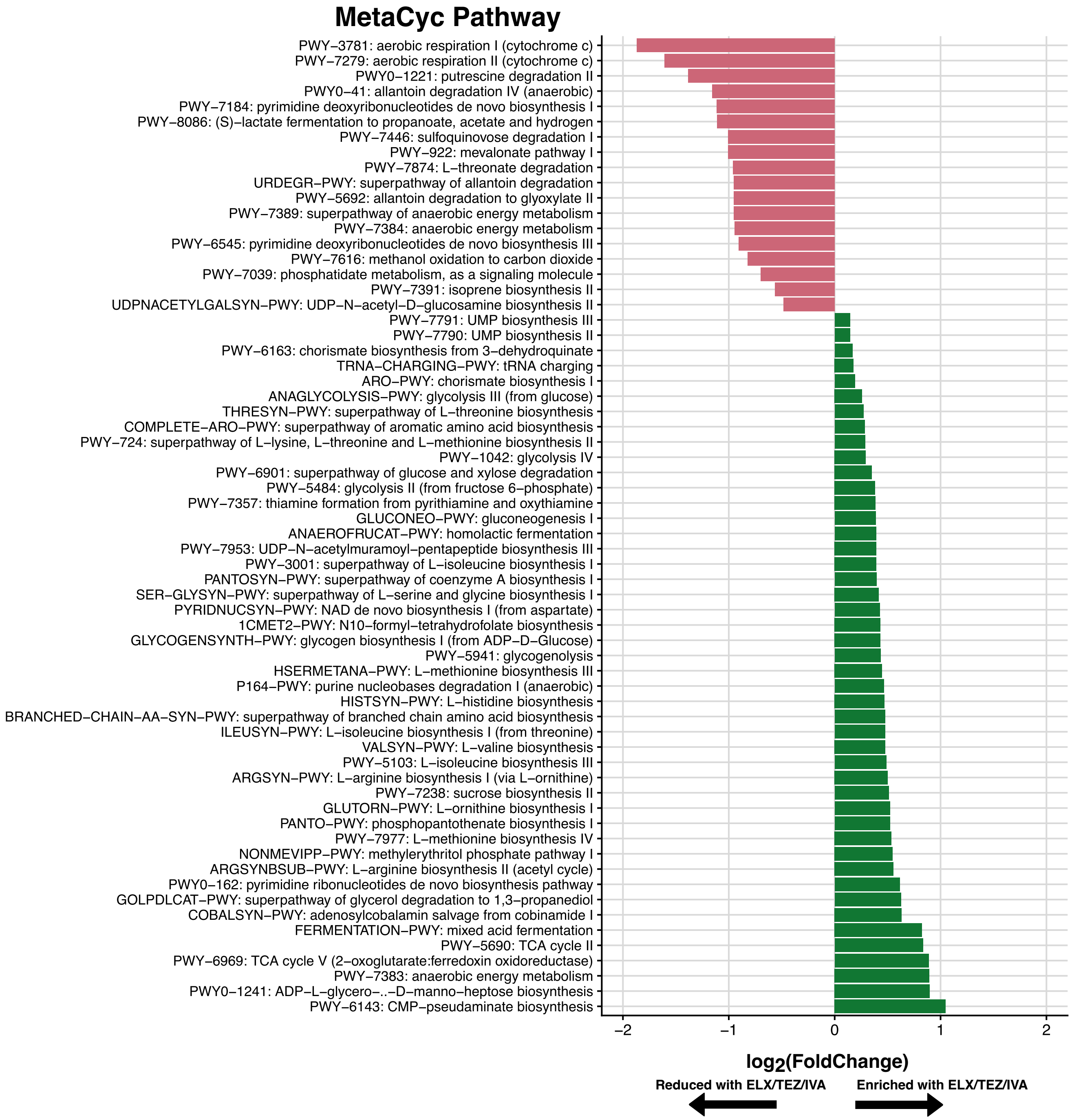

Supplementary Figure S7. Differentially abundant MetaCyc pathways following MaAsLin2 multivariable association modeling, using ELX/TEZ/IVA status, age, and recent antibiotics as fixed effects in the model. Only pathways with FDR<0.25 are depicted.
