# Supplementary material for: Longitudinal profiling of the intestinal microbiome in children with cystic fibrosis treated with elexacaftor-tezacaftor-ivacaftor
Source: mBio. 2024 Jan 26;15(2):e01935-23. doi: 10.1128/mbio.01935-23 (PMC10865789; doi:10.1128/mbio.01935-23)
Supplement: Supplemental Methods — Representative code for all bioinformatic processing and statistical analyses. [file mbio.01935-23-s0003.pdf]

CF-GI-Microbiome-ELX-TEZ-IVA

Seth Reasoner

2023-05-15

Contents

Introduction 1

Data Availability 2

Bioinformatic processing of sequences 2

Trimming raw reads using fastq-mcf . . . 2

Taxonomic profiling using MetaPhlAn4 . . . 2

Functional profiling using HUMAnN3 . . . 2

Identification of Antibiotic Resistance Genes with ShortBRED . . . 3

Statistical Analyses 4

Calculating Diversity Metrics . . . 4

Calculating Microbial Dysbiosis Index . . . 7

Differential Abundance Testing of Microbial Species . . . 8

Differential Abundance Testing of All Taxonomic Ranks . . . 9

Differential Abundance Testing of Antibiotic Resistance Genes (ARGs) . . . 9

Differential Abundance Testing of KEGG Orthology Groups . . . 10

Differential Abundance Testing of MetaCyc Pathways . . . 10

R Session Information 11

Introduction

This file includes documentation detailing the code used for bioinformatics and statistical analysis accompanying the manuscript ***Longitudinal Profiling of the Intestinal Microbiome in Children with Cystic Fibrosis Treated with Elexacaftor-Tezacaftor-Ivacaftor***. In this study, stool samples were collected from 39 children with cystic fibrosis (CF) before and after treatment with the CFTR modulator ELX-TEZ-IVA. A total of 114 stool samples were collected and subjected to shotgun metagenomic sequencing. Samples are named P#T#; the number following the "P" refers to the subject ID (#1-39), the number following the "T" refers to the timepoint from which the sample was collected (#1-4) (see Figure 1B). We used biobakery3 workflows (PMID: 33944776) to taxonomically and functionally profile the samples. This document provides representative code to reproduce the processing and statistical analyses presented in this manuscript. (**n.b. ELX-TEZ-IVA will be referred to by its brand name 'Trikafta' within the code for simplicity of variable calling**).

Data Availability

Raw sequence data can be accessed via NCBI BioProject PRJNA948536. Processed data tables are available within the data subdirectory of this repository and within Supplementary Dataset 1.

Bioinformatic processing of sequences

Bioinformatic processing of raw sequencing reads was conducted via the command line with the code in the following sections.

Trimming raw reads using fastq-mcf

- o: output files
- D: remove duplicate reads with # identical bases
- k: sKew percentage
- q: quality threshold for base removal
- l: minimum remaining sequence length

```
# perform adapter trimming and quality trimming/filtering for each sample

fastq-mcf \
-o ${SAMPLE_NAME}_R1.trimmed.fastq -o ${SAMPLE_NAME}_R2.trimmed.fastq \
-D 0 -k 0 -q 5 -l 10 illumina_adaptors.fa \
${SAMPLE_NAME}_R1.fastq ${SAMPLE_NAME}_R2.fastq #input files
```

Taxonomic profiling using MetaPhlAn4

Metaphlan4 is an open source software available via `https://github.com/biobakery/MetaPhlAn`.

```
metaphlan \
${SAMPLE_NAME}_R1.trimmed.fastq,${SAMPLE_NAME}_R2.trimmed.fastq
--bowtie2out metagenome.bowtie2.bz2 \
-t rel_ab \
--nproc 20 \
--input_type fastq \
--bowtie2db metaphlan4_db \
-o Taxonomy/${SAMPLE_NAME}_profiled_metagenome.txt

# merge individual sample files
merge_metaphlan_tables.py \
Taxonomy/*_profiled_metagenome.txt > Taxonomy/metaphlan_combined_taxonomy.tsv

# limit taxonomy to species level
grep -E "(^taxonomy)|(s__)" metaphlan_combined_taxonomy.tsv | grep -v "t__" |
  sed "s/.*s__//g" > metaphlan_species_relativeabundance.tsv
```

Functional profiling using HUMAnN3

HUMAnN3 is an open source software available via `http://huttenhower.sph.harvard.edu/humann`. Functional profiling was conducted with the UniRef database with sequences grouped at 90% identity. UniRef90 gene families were regrouped into more interpretable orthology groups (KEGG) and pathways (MetaCyc).

```
humann \
-i ${SAMPLE_NAME}_combined.trimmed.fastq \ # using concatenated trimmed reads
-o FunctionalProfiling  \
--threads 20 \
--search-mode uniref90 \
--nucleotide-database /path/to/mpa_vJan21_CHOCOPhlAnSGB_202103 \
--protein-database /path/to/uniref90_annotated_v201901b_full \
--metaphlan-options= "--bowtie2db /path/to/metaphlan4"

# merge MetaCyc pathway abundances across samples into single file
humann_join_tables \
-i FunctionalProfiling \
-o merged_pathabundance.tsv \
--file_name _pathabundance.tsv

# normalize pathway abundance to counts per million (cpm)
humann_renorm_table \
-i merged_pathabundance.tsv \
-o humann_metacyc_stratified.tsv \
--units cpm

# stratified output includes species contributing to each functional annotation
# remove stratified taxonomic output, limit to community
grep -v "|" humann_metacyc_stratified.tsv > humann_metacyc_pathwayabundance.tsv

# map UniRef90 groups (gene families output from HUMAnN3) to KEGG Orthology (KO) Groups
humann_regroup_table \
-i ${SAMPLE_NAME}.combined.trimmed_genefamilies.tsv \
-c /path/to/utility_mapping/map_ko_uniref90.txt.gz \
-o FunctionalProfiling/${SAMPLE_NAME}_KOcounts.tsv

# merge KO Groups across samples into single file
humann_join_tables \
-i FunctionalProfiling \
-o merged_KOcounts.tsv \
--file_name _KOcounts.tsv

# normalize KO abundances to counts per million (cpm)
humann_renorm_table \
-i merged_KOcounts.tsv \
-o humann_KO_abundance_stratified.tsv \
--units cpm

# remove stratified taxonomic output, limit to community
grep -v "|" humann_KO_abundance_stratified.tsv > humann_KO_abundance.tsv
```

Identification of Antibiotic Resistance Genes with ShortBRED

ShortBRED is an open source software available via `https://huttenhower.sph.harvard.edu/shortbred/`. We used ShortBRED to quantify antibiotic resistance genes with high specificity. We used the Comprehensive Antibiotic Resistance Database (CARD v. 2017, PMID: 27789705) as the reference database.

```
shortbred_quantify.py \
--markers ShortBRED_CARD_2017_markers.faa \ #database of antibiotic resistance gene markers
```

```
--threads 20 \
--wgs ${SAMPLE_NAME}_R1.trimmed.fastq ${SAMPLE_NAME}_R2.trimmed.fastq \
--results trimmed_shortbred_results.tsv \
--tmp trimmed
```

Statistical Analyses

Calculating Diversity Metrics

```
# load requisite packages
library(tidyverse)
library(vegan)
library(rbiom)
library(ape)
library(here)
library(ggpubr)

# load species table
# (metaphlan_species_relativeabundance.tsv)
species <- read.table("https://raw.githubusercontent.com/reaset41/CF-GI-Microbiome-ELX-TEZ-IVA/main/data/me
    header = TRUE, row.names = 1)

# load metadata
metadata <- read.csv("https://raw.githubusercontent.com/reaset41/CF-GI-Microbiome-ELX-TEZ-IVA/main/data/met
    row.names = 1, header = FALSE)

# calculate richness (observed species) and Shannon Index
# from species table

shannon <- diversity(t(species), index = "shannon")
observed_species <- colSums(species != 0)
alpha_diversity <- as.data.frame(cbind(shannon, observed_species))

## add metadata for graphs
alpha_diversity$Trikafta <- t(metadata[10, ])
alpha_diversity$RecentAbx_binary <- t(metadata[30, ])

## alpha diversity graphs with respect to Trikafta status
GroupColors <- c("#CC6677", "#117733")
# Figure 2A
shannon_trikafta <- ggplot(alpha_diversity, aes(x = reorder(Trikafta,
    +shannon), y = shannon, color = Trikafta)) + geom_boxplot(outlier.shape = NA,
    size = 1, width = 0.75) + theme_classic() + geom_jitter(width = 0.15,
    alpha = 0.4, size = 3) + xlab("") + ylim(0, 4.5) + scale_color_manual(values = GroupColors) +
    theme(axis.text = element_text(size = 18), axis.title = element_text(size = 16),
        panel.border = element_rect(colour = "black", fill = NA,
            size = 1)) + stat_compare_means(method = "wilcox.test")

# Figure 2B
observed_trikafta <- ggplot(alpha_diversity, aes(x = reorder(Trikafta,
    +observed_species), y = observed_species, color = Trikafta)) +
    geom_boxplot(outlier.shape = NA, size = 1, width = 0.75) +
    theme_classic() + geom_jitter(width = 0.15, alpha = 0.4,
```

```
    size = 3) + xlab("") + ylim(0, 200) + scale_color_manual(values = GroupColors) +
    theme(axis.text = element_text(size = 18), axis.title = element_text(size = 16),
        panel.border = element_rect(colour = "black", fill = NA,
            size = 1)) + stat_compare_means(method = "wilcox.test")

## alpha diversity graphs with respect to recent antibiotic
## exposure
AbxColors <- c("#003f5c", "#FFA600")
# Figure 2C
shannon_recentAbx <- ggplot(alpha_diversity, aes(x = reorder(RecentAbx_binary,
    +shannon), y = shannon, color = RecentAbx_binary)) + geom_boxplot(outlier.shape = NA,
    size = 1, width = 0.75) + theme_classic() + geom_jitter(width = 0.15,
    alpha = 0.4, size = 3) + xlab("") + ylim(0, 4.5) + scale_color_manual(values = AbxColors) +
    theme(axis.text = element_text(size = 18), axis.title = element_text(size = 16),
        panel.border = element_rect(colour = "black", fill = NA,
            size = 1)) + stat_compare_means(method = "wilcox.test")

# Figure 2D
observed_recentAbx <- ggplot(alpha_diversity, aes(x = reorder(RecentAbx_binary,
    +observed_species), y = observed_species, color = RecentAbx_binary)) +
    geom_boxplot(outlier.shape = NA, size = 1, width = 0.75) +
    theme_classic() + geom_jitter(width = 0.15, alpha = 0.4,
    size = 3) + xlab("") + ylim(0, 200) + scale_color_manual(values = AbxColors) +
    theme(axis.text = element_text(size = 18), axis.title = element_text(size = 16),
        panel.border = element_rect(colour = "black", fill = NA,
            size = 1)) + stat_compare_means(method = "wilcox.test")


# calculate beta diversity

## Bray-Curtis using species file from Metaphlan processing
species_matrix <- species %>%
    as.matrix() %>%
    t()

bray_dist_mat <- vegdist(species_matrix, method = "bray")

pca_bray <- cmdscale(bray_dist_mat, k = (nrow(species_matrix) -
    1), eig = TRUE)

pca_df_bray <- tibble(PC1 = pca_bray$points[, 1], PC2 = pca_bray$points[,
    2])

pca_df_bray$SampleName <- t(metadata[1, ])
pca_df_bray$Trikafta <- t(metadata[10, ])
pca_df_bray$RecentAbx_binary <- t(metadata[30, ])

## Bray-Curtis plot with respective to Trikafta status
## (Figure S2C)
GroupColors <- c("#CC6677", "#117733")
bray_Trikafta <- ggplot(pca_df_bray, aes(x = PC1, y = PC2, color = Trikafta)) +
    geom_point(size = 2) + stat_ellipse(aes(group = Trikafta)) +
    scale_color_manual(values = GroupColors) + theme_classic() +
    theme(axis.text = element_text(size = 16), axis.title = element_text(size = 16),
        panel.border = element_rect(colour = "black", fill = NA,
            size = 1))
```

```
adonis2(bray_dist_mat ~ Trikafta, data = pca_df_bray, permutations = 9999)
bd_bray_trikafta <- betadisper(bray_dist_mat, pca_df_bray$Trikafta)
anova(bd_bray_trikafta)

## Bray-Curtis plot with respective to recent antibiotic
## exposure (Figure S2E)
AbxColors <- c("#003f5c", "#FFA600")

bray_Abx <- ggplot(pca_df_bray, aes(x = PC1, y = PC2, color = RecentAbx_binary)) +
    geom_point(size = 2) + stat_ellipse(aes(group = RecentAbx_binary)) +
    scale_color_manual(values = AbxColors) + theme_classic() +
    theme(axis.text = element_text(size = 16), axis.title = element_text(size = 16),
        panel.border = element_rect(colour = "black", fill = NA,
            size = 1))

adonis2(bray_dist_mat ~ RecentAbx_binary, data = pca_df_bray,
    permutations = 9999)
bd_bray_Abx <- betadisper(bray_dist_mat, pca_df_bray$RecentAbx_binary)
anova(bd_bray_Abx)

## calculate weighted unifrac distance matrix using
## modified code from metaphlan

mpa_infile <- read.table("https://github.com/reaset41/CF-GI-Microbiome-ELX-TEZ-IVA/raw/main/data/metaphlan_
    header = TRUE, row.names = 1)

tree_file <- "mpa_v30_CHOCOPhlAn_201901_species_tree.nwk"
# this tree file is available from Metaphlan
outfile <- "unifrac.tsv"

mpa_table <- read.table(mpa_infile, comment.char = "#", sep = "\t",
    header = TRUE)
mpa_table <- mpa_table[grep("s__", mpa_table[, 1]), ]
mpa_table[, 1] <- gsub(".+\\|s__", "", mpa_table[, 1])
rownames(mpa_table) <- mpa_table[, 1]
mpa_table <- mpa_table[, -1]

mpa_tree <- ape::read.tree(tree_file)
mpa_tree$tip.label <- gsub(".+\\|s__", "", mpa_tree$tip.label)

filt_tree <- ape::keep.tip(mpa_tree, intersect(rownames(mpa_table),
    mpa_tree$tip.label))
filt_mpa_table <- mpa_table[filt_tree$tip.label, ]/100
rbiom_distmat <- rbiom::unifrac(as.matrix(filt_mpa_table), weighted = TRUE,
    tree = filt_tree)
write.table(as.matrix(rbiom_distmat), outfile, sep = "\t", quote = FALSE)

## weighted unifrac plot with respect to Trikafta status
## (Figure S2D)

pca_unifrac <- cmdscale(rbiom_distmat, k = (nrow(species_matrix) -
    1), eig = TRUE)
pca_df_unifrac <- tibble(PC1 = pca_unifrac$points[, 1], PC2 = pca_unifrac$points[,
    2])

pca_df_unifrac$SampleName <- t(metadata[1, ])
```

```
pca_df_unifrac$Trikafta <- t(metadata[10, ])

unifrac_Trikafta <- ggplot(pca_df_unifrac, aes(x = PC1, y = PC2,
    color = Trikafta)) + geom_point(size = 2) + stat_ellipse(aes(group = Trikafta)) +
    scale_color_manual(values = GroupColors) + theme_classic() +
    theme(axis.text = element_text(size = 16), axis.title = element_text(size = 16),
        panel.border = element_rect(colour = "black", fill = NA,
            size = 1))

adonis2(rbiom_distmat ~ Trikafta, data = pca_df_unifrac, permutations = 9999)
bd_unifrac_trikafta <- betadisper(rbiom_distmat, pca_df_unifrac$Trikafta)
anova(bd_unifrac_trikafta)
```

Calculating Microbial Dysbiosis Index

```
# load full taxonomy table
# (metaphlan_combined_taxonomy.tsv)
full_taxonomy <- read.table("https://github.com/reaset41/CF-GI-Microbiome-ELX-TEZ-IVA/raw/main/data/metaphl
    header = TRUE, row.names = 1)

# the microbial dysbiosis index was originally published in
# 2014 (PMID:24629344)

## Taxa in the numerator (increased in treatment naive
## Crohn's disease): Enterobacteriaceae, Pasteurellaceae,
## Fusobacteriaceae, Neisseriaceae, Veillonellaceae,
## Gemellaceae

Enterobacteriaceae <- full_taxonomy["k__Bacteria|p__Proteobacteria|c__Gammaproteobacteria|o__Enterobacteral
    ]

Pasteurellaceae <- full_taxonomy["k__Bacteria|p__Proteobacteria|c__Gammaproteobacteria|o__Pasteurellales|f_
    ]

Fusobacteriaceae <- full_taxonomy["k__Bacteria|p__Fusobacteria|c__Fusobacteriia|o__Fusobacteriales|f__Fusob
    ]

Neisseriaceae <- full_taxonomy["k__Bacteria|p__Proteobacteria|c__Betaproteobacteria|o__Neisseriales|f__Neis
    ]

Veillonellaceae <- full_taxonomy["k__Bacteria|p__Firmicutes|c__Negativicutes|o__Veillonellales|f__Veillonel
    ]

Gemellaceae <- replace(full_taxonomy["k__Bacteria|p__Firmicutes|c__Bacilli|o__Bacillales|f__Gemellaceae",
    ], is.na(full_taxonomy["k__Bacteria|p__Firmicutes|c__Bacilli|o__Bacillales|f__Gemellaceae",
    ]), 0)

## Taxa in the denominator (decreased in treatment naive
## Crohn's disease): Bacteroidales, Clostridiales
## (excluding Veillonellaceae),Erysipelotrichaceae, and
## Bifidobacteriaceae

Bacteroidales <- full_taxonomy["k__Bacteria|p__Bacteroidetes|c__Bacteroidia|o__Bacteroidales",
    ]
```

```
Clostridiales <- full_taxonomy["k__Bacteria|p__Firmicutes|c__Clostridia|o__Clostridiales",
    ] - Veillonellaceae - full_taxonomy["k__Bacteria|p__Firmicutes|c__Negativicutes|o__Veillonellales|f__Ve
    ]

Erysipelotrichaceae <- full_taxonomy["k__Bacteria|p__Firmicutes|c__Erysipelotrichia|o__Erysipelotrichales|f
    ]

Bifidobacteriaceae <- full_taxonomy["k__Bacteria|p__Actinobacteria|c__Actinobacteria|o__Bifidobacteriales|f
    ]

## aggregate taxa together
numerator_rows <- rbind(Enterobacteriaceae, Pasteurellaceae,
    Fusobacteriaceae, Neisseriaceae, Veillonellaceae, Gemellaceae)
denominator_rows <- rbind(Bacteroidales, Clostridiales, Erysipelotrichaceae,
    Bifidobacteriaceae)

num <- colSums(numerator_rows)
denom <- colSums(denominator_rows)

# MD Index: log[(numerator)/(denominator)]
MD <- as.data.frame(cbind(num, denom))

MD$index <- log10(MD$num/MD$denom)

## add metadata for graph
MD$Trikafta <- t(metadata[10, ])
MD$RecentAbx_binary <- t(metadata[30, ])

## microbial dysbiosis index by Trikafta status (Figure 4B)
MDindex_Trikafta <- ggplot(MD, aes(x = Trikafta, y = index, color = Trikafta)) +
    geom_boxplot(outlier.shape = NA, size = 1, width = 0.75) +
    theme_classic() + geom_jitter(width = 0.15, alpha = 0.4,
    size = 3) + xlab("") + ylim(-3, 1) + scale_color_manual(values = GroupColors) +
    theme(axis.text = element_text(size = 18), axis.title = element_text(size = 16),
        panel.border = element_rect(colour = "black", fill = NA,
            size = 1)) + stat_compare_means(method = "wilcox.test")

## microbial dysbiosis index by recent antibiotic exposure
## (Figure 4C)
MDindex_RecentAbx <- ggplot(MD, aes(x = reorder(RecentAbx_binary,
    index), y = index, color = RecentAbx_binary)) + geom_boxplot(outlier.shape = NA,
    size = 1, width = 0.75) + theme_classic() + geom_jitter(width = 0.15,
    alpha = 0.4, size = 3) + xlab("") + ylim(-3, 1) + scale_color_manual(values = AbxColors) +
    theme(axis.text = element_text(size = 18), axis.title = element_text(size = 16),
        panel.border = element_rect(colour = "black", fill = NA,
            size = 1)) + stat_compare_means(method = "wilcox.test")
```

Differential Abundance Testing of Microbial Species

```
# load requisite libraries
library(Maaslin2)

# load metadata with sample names as headers
metadata_maaslin2 <- read.csv("https://raw.githubusercontent.com/reaset41/CF-GI-Microbiome-ELX-TEZ-IVA/main
```

```
    header = TRUE, row.names = 1)

# use species table from above
# (metaphlan_species_relativeabundance.tsv)

species_maaslin2 <- Maaslin2(input_data = species, input_metadata = metadata_maaslin2,
    min_prevalence = 0.1, min_abundance = 0, normalization = "NONE",
    transform = "LOG", output = "species_maaslin2", fixed_effects = c("Trikafta",
        "RecentAbx_binary", "Age"), reference = c("Trikafta,N",
        "RecentAbx_binary,N"), random_effects = "PatientNumber",
    analysis_method = "LM", correction = "BH", max_significance = 0.1,
    plot_heatmap = FALSE, plot_scatter = FALSE)


# these results (all_results.tsv) make up Table S2
write.csv(species_maaslin2$results, file = "Table S2.csv", row.names = FALSE)

# these results make up Table S3 (limited to species that
# change with respect to Trikafta)
species_results <- species_maaslin2$results
Table_S3 <- species_results[species_results$metadata == "Trikafta" &
    species_results$qval < 0.1, ]

write.csv(Table_S3, file = "Table S3.csv", row.names = FALSE)
```

Differential Abundance Testing of All Taxonomic Ranks

```
# use full taxonomy table (metaphlan_combined_taxonomy.tsv)
full_taxonomy

taxonomy_maaslin2 <- Maaslin2(input_data = full_taxonomy, input_metadata = metadata_maaslin2,
    min_prevalence = 0.1, min_abundance = 0, normalization = "NONE",
    transform = "LOG", output = "taxonomy_maaslin2", fixed_effects = c("Trikafta",
        "RecentAbx_binary", "Age"), reference = c("Trikafta,N",
        "RecentAbx_binary,N"), random_effects = "PatientNumber",
    analysis_method = "LM", correction = "BH", max_significance = 0.1,
    plot_heatmap = FALSE, plot_scatter = FALSE)

# these results make up Table S4 and contribute to Figures
# 3B & S4
write.csv(taxonomy_maaslin2$results, file = "Table S4.csv", row.names = FALSE)
```

Differential Abundance Testing of Antibiotic Resistance Genes (ARGs)

```
# load ARG data table (shortbred_ARG-rpkm.csv)
ARG_input_data <- read.csv("https://raw.githubusercontent.com/reaset41/CF-GI-Microbiome-ELX-TEZ-IVA/main/da
    row.names = 1, header = TRUE)

ARG_maaslin2 <- Maaslin2(input_data = ARG_input_data, input_metadata = metadata_maaslin2,
    min_prevalence = 0.1, min_abundance = 0, normalization = "NONE",
    transform = "LOG", output = "output_ARG", fixed_effects = c("Trikafta",
        "RecentAbx_binary", "Age"), reference = c("Trikafta,N",
```

```r
        "RecentAbx_binary,N"), random_effects = "PatientNumber",
    analysis_method = "LM", correction = "BH", max_significance = 0.25,
    plot_heatmap = FALSE, plot_scatter = FALSE)

# these results make up Table S5 and are used in Figure S3D
# & S3F
write.csv(ARG_maaslin2$results, file = "Table S5.csv", row.names = FALSE)
```

Differential Abundance Testing of KEGG Orthology Groups

```r
# load KEGG Orthologs file (humann_KO_abundance.tsv)
KO_groups <- read.table("https://raw.githubusercontent.com/reaset41/CF-GI-Microbiome-ELX-TEZ-IVA/main/data/
    header = TRUE, sep = "\t", row.names = 1, stringsAsFactors = FALSE)

KEGG_maaslin2 <- Maaslin2(input_data = KO_groups, input_metadata = metadata_maaslin2,
    min_prevalence = 0.1, min_abundance = 0, normalization = "NONE",
    transform = "LOG", output = "output_KEGG", fixed_effects = c("Trikafta",
        "RecentAbx_binary", "Age"), reference = c("Trikafta,N",
        "RecentAbx_binary,N"), random_effects = "PatientNumber",
    analysis_method = "LM", correction = "BH", max_significance = 0.25,
    plot_heatmap = FALSE, plot_scatter = FALSE)

# these results make up Table S6
write.csv(KEGG_maaslin2$results, file = "Table S6.csv", row.names = FALSE)

# these results make up Table S7 (limited to groups that
# change with respect to Trikafta)
KEGG_results <- KEGG_maaslin2$results
Table_S7 <- KEGG_results[KEGG_results$metadata == "Trikafta" &
    KEGG_results$qval < 0.25, ]

write.csv(Table_S7, file = "Table S7.csv", row.names = FALSE)
```

Differential Abundance Testing of MetaCyc Pathways

```r
# load MetaCyc Pathways file
# (humann_metacyc_pathwayabundance.tsv)
Metacyc_pathways <- read.table("https://raw.githubusercontent.com/reaset41/CF-GI-Microbiome-ELX-TEZ-IVA/mai
    header = TRUE, sep = "\t", row.names = 1, stringsAsFactors = FALSE)

Metacyc_maaslin2 <- Maaslin2(input_data = Metacyc_pathways, input_metadata = metadata_maaslin2,
    min_prevalence = 0.1, min_abundance = 0, normalization = "NONE",
    transform = "LOG", output = "output_metacyc", fixed_effects = c("Trikafta",
        "RecentAbx_binary", "Age"), reference = c("Trikafta,N",
        "RecentAbx_binary,N"), random_effects = "PatientNumber",
    analysis_method = "LM", correction = "BH", max_significance = 0.25,
    plot_heatmap = FALSE, plot_scatter = FALSE)

# these results make up Table S8
write.csv(Metacyc_maaslin2$results, file = "Table S8.csv", row.names = FALSE)

# these results make up Table S9 (limited to pathways that
```

```r
# change with respect to Trikafta)
MetaCyc_results <- Metacyc_maaslin2$results
Table_S9 <- MetaCyc_results[MetaCyc_results$metadata == "Trikafta" &
    MetaCyc_results$qval < 0.25, ]

write.csv(Table_S9, file = "Table S9.csv", row.names = FALSE)
```

R Session Information

```r
sessionInfo()
```

```
## R version 4.3.1 (2023-06-16)
## Platform: aarch64-apple-darwin20 (64-bit)
## Running under: macOS Monterey 12.5
##
## Matrix products: default
## BLAS:   /Library/Frameworks/R.framework/Versions/4.3-arm64/Resources/lib/libRblas.0.dylib
## LAPACK: /Library/Frameworks/R.framework/Versions/4.3-arm64/Resources/lib/libRlapack.dylib;  LAPACK versi
##
## locale:
## [1] en_US.UTF-8/en_US.UTF-8/en_US.UTF-8/C/en_US.UTF-8/en_US.UTF-8
##
## time zone: America/Chicago
## tzcode source: internal
##
## attached base packages:
## [1] stats     graphics  grDevices utils     datasets  methods   base
##
## loaded via a namespace (and not attached):
##  [1] compiler_4.3.1    fastmap_1.1.1     cli_3.6.1         formatR_1.14
##  [5] tools_4.3.1       htmltools_0.5.6.1 rstudioapi_0.15.0 yaml_2.3.7
##  [9] rmarkdown_2.25    knitr_1.44        xfun_0.40         digest_0.6.33
## [13] rlang_1.1.1       evaluate_0.22
```
